# Benefits of, Barriers to, and Needs for an Artificial Intelligence–Powered Medication Information Voice Chatbot for Older Adults: Interview Study With Geriatrics Experts

**DOI:** 10.2196/32169

**Published:** 2022-04-28

**Authors:** Meghana Gudala, Mary Ellen Trail Ross, Sunitha Mogalla, Mandi Lyons, Padmavathy Ramaswamy, Kirk Roberts

**Affiliations:** 1 School of Biomedical Informatics University of Texas Health Science Center at Houston Houston, TX United States; 2 Cizik School of Nursing University of Texas Health Science Center at Houston Houston, TX United States

**Keywords:** medication information, chatbot, older adults, technology capabilities, mobile phone

## Abstract

**Background:**

One of the most complicated medical needs of older adults is managing their complex medication regimens. However, the use of technology to aid older adults in this endeavor is impeded by the fact that their technological capabilities are lower than those of much of the rest of the population. What is needed to help manage medications is a technology that seamlessly integrates within their comfort levels, such as artificial intelligence agents.

**Objective:**

This study aimed to assess the benefits, barriers, and information needs that can be provided by an artificial intelligence–powered medication information voice chatbot for older adults.

**Methods:**

A total of 8 semistructured interviews were conducted with geriatrics experts. All interviews were audio-recorded and transcribed. Each interview was coded by 2 investigators (2 among ML, PR, METR, and KR) using a semiopen coding method for qualitative analysis, and reconciliation was performed by a third investigator. All codes were organized into the benefit/nonbenefit, barrier/nonbarrier, and need categories. Iterative recoding and member checking were performed until convergence was reached for all interviews.

**Results:**

The greatest benefits of a medication information voice-based chatbot would be helping to overcome the vision and dexterity hurdles experienced by most older adults, as it uses voice-based technology. It also helps to increase older adults’ medication knowledge and adherence and supports their overall health. The main barriers were technology familiarity and cost, especially in lower socioeconomic older adults, as well as security and privacy concerns. It was noted however that technology familiarity was not an insurmountable barrier for older adults aged 65 to 75 years, who mostly owned smartphones, whereas older adults aged >75 years may have never been major users of technology in the first place. The most important needs were to be usable, to help patients with reminders, and to provide information on medication side effects and use instructions.

**Conclusions:**

Our needs analysis results derived from expert interviews clarify that a voice-based chatbot could be beneficial in improving adherence and overall health if it is built to serve the many medication information needs of older adults, such as reminders and instructions. However, the chatbot must be usable and affordable for its widespread use.

## Introduction

Older adults (defined here as those aged ≥65 years) have multiple chronic diseases [1] and consequently take far more medications than the average individual [2]. One study estimated that 39% of older adults had ≥5 concurrent prescriptions, and this number had tripled from that 20 years ago [3]. Complicating this situation further is the reduced mental capacity of older adults [4]. This means that the cognitive burden of keeping track of medications is well beyond the capabilities of many older adults, resulting in poor adherence [5] and consequently affecting health. Older adults often rely on caregivers and low-tech solutions such as pillboxes, but technology is often seen as a barrier for older adults. However, as Olsen et al [6] described, although the range and frequency of technology use among older adults may be less than that of younger adults, the capability still exists for certain types of technologies. A technology that relies on their existing knowledge and experience has the capacity for widespread adoption. The primary significance of this study is that it gauges the potential incorporation of a tool capable of improving medication understanding and adherence among older adults using a technology that mimics everyday human behavior of voice conversations: a chatbot. Indeed, voice-based chatbots have already been seen as a potential aid for older adults [7] and early-stage implementations of such systems for medication information exist [8], although these are clearly not in common use and many barriers remain to their successful adoption. To understand the capabilities of a medication information chatbot for older adults, we conducted a qualitative needs analysis using interviews with a wide range of geriatrics experts. The interviews were limited to geriatrics experts, as this was felt to be the best way to engage experts, whereas actual older adults would be better engaged through separate simulation-based studies. We then analyzed the experts’ beliefs about the capabilities of older adults with such a chatbot with regard to managing their medications and what their medication information needs were.

The subject of older adults and chatbots has been explored previously in several studies. Martin-Hammond et al [9] assessed the general attitudes of older adults toward intelligent assistants (IAs; a class of agents that includes the voice-based chatbots studied here), finding them very positive. The study participants viewed IAs as great opportunities that could facilitate collaboration between themselves and their caregivers. They also considered IAs to be very useful in providing recommendations and alerts for serious illness. However, they preferred the assistants to be more flexible so that all sections of older adults could use them, including those with low technical resources and skills. Moreover, having an interactive IA that could mimic natural interactions regarding health information was viewed as more desirable. Chatbots have also been proposed to improve Wikipedia use and editing to circumvent the steep learning curve for older adults [10]. A study on the use of chatbots for psychological support purposes showed that they could be useful for resolving problems and lowering distress [11]. The chatbots were designed to mimic therapists, and the agent’s usability was associated with their helpfulness. Overall, these studies suggest that well-designed intelligent agents would be well-received by older adults despite the technology not currently being used.

With regard to medication information, older adults find pharmacists most useful, both for managing their medication lists and educating them with instructions [12]. A study was conducted to understand older adults’ expectations and requirements for a personal health application that could meet their information needs [13]. The interviewed patients and caregivers reported the following as top requirements: (1) having the capability to disclose medication side effects and interactions in a clear and easy manner, (2) being able to connect their providers and pharmacies, and (3) being able to share their medication information with other providers. Another study noted an interesting aspect of interviewing patients upon discharge from the hospital. Although having information about their medications, alternative treatment options, and side effects were the most important needs, some patients did not actually want to fully understand the medications and their side effects, as they were afraid that knowing them might change their attitude toward the medications [14]. This suggests that although a chatbot could be beneficial, it should be designed to not overwhelm patients with details beyond their grasp. For instance, even common medications have a long list of side effects that patients are unaware of, so providing information on a long list of rare side effects for a new medication may give the patient the false impression that the medication is dangerous relative to the medications they already regularly take.

The role of caregivers, such as home care nurses, was explored in a study [15], wherein the challenges of the transition of care between various settings were studied. Whenever older adults were moved from hospitals to home or nursing care, changes to their medications and administration instructions would change. In such cases, home care nurses played a big role in helping the older adults adapt and follow the new medication changes and manage medication compliance. This highlights the importance of caregivers and their roles in managing the overall health of older adults. This suggests that voice-based chatbots cannot fully replace existing human interactions for medical information, and should thus focus on supplementing existing relationships and information sources. One of the aspects of medication management for older adults was their trust in resources. A survey conducted to identify the resources that were trusted more [16] for health information needs showed that older adults placed living resources higher than nonliving resources. The top priorities for seeking information were health care providers and pharmacists.

Overall, the use of health applications and computer assistants in older adults to assist with their medication management and self-care has been an area of interest [17-21]. We continue to explore this area in this study to make medication management easier and safer in older adults. Specifically, this paper’s contribution is to summarize the beliefs of geriatrics experts on the benefits, barriers, and needs of such a voice-based medication information chatbot. Using structured interviews and a rigorous qualitative coding process, we identified key themes from this group.

## Methods

### Overview

The needs analysis collected data on high-level needs to assess the feasibility and system requirements for an artificial intelligence (AI)-powered medication information voice chatbot for older adults. For the purposes of this study, we define an *AI-powered medication information chatbot* as an automated dialogue agent that integrates human language understanding to provide evidence-based information about prescription medications. Data were collected through semistructured interviews with geriatrics experts, including physicians, nurses, researchers, and pharmacists. A total of 8 interviews were conducted, each with at least 1 of the investigator coauthors, of whom all were nursing faculty members (ML, PR, and METR). All interviews were recorded, transcribed, and deidentified. Manual coding was performed using the 3 nursing experts as well as an expert in AI and natural language processing (KR), with each interview being coded by 2 investigators (2 of ML, PR, METR, and KR). Intercoder agreement was noted to evaluate the reliability of the analyzed feedback.

### Study Funding

This research was funded by the UTHealth School of Nursing through an Aging in Place seed grant.

### Ethics Approval

The study was approved by the UTHealth Center for the Protection of Human Subjects (approval number: HSC-SBMI-20-0526).

### Data Collection

We collected data using semistructured interviews with 8 participants selected to ensure a diversity of geriatrics expertise, including physicians, nurses, pharmacists, and researchers. Most of the interview participants were from Houston, Texas. Interviews were conducted via WebEx by 1 or 2 of the coauthors (ML, PR, and METR). They consisted of open-ended questions regarding older adults, their view on technology, use, limitations, barriers, what would older adults need most with regard to medication information, and what could be provided using technology (Textbox 1 describes the open-ended questions). The focus was on both the current generation of older adults aged 65 to 75 years (at the time of the study, this represents those born roughly between 1945 and 1955) as well as adults relatively soon to join this group, individuals aged 55 to 65 years (born between 1955 and 1965). It was assumed that adults aged ≥75 years (born before 1945) may have different needs, both from an aging perspective and a technology familiarity perspective (eg, almost all adults ≤75 years have owned smartphones). For the purpose of the interview, it was assumed that the term medication applies to prescription medications that have been prescribed within the past 6 months, as opposed to medication, and one may expect to have been using long-term. Each interview lasted 30 to 40 minutes. All interviewees were informed that their participation in the interview would not be revealed to anyone beyond the investigators, and that their responses would be kept in strictest confidence. If the interviewee was specialized in any specific disease condition and wanted to limit their responses to that area, they were encouraged to do so. However, if the interviewees had a generalized idea, they were welcomed to share those views as well. The interviews were audio-recorded, and later, a research assistant transcribed the content verbatim to analyze the responses in text format.

**Common framework of open-ended questions used for the semistructured interviews.**

**Open-ended questions**

- Question 1: What are older adults’ comfort level and capabilities with the use of technology in general?
- Question 2: What are older adults’ comfort level and capabilities regarding the use of voice-based technology like Alexa, Siri, etc?
- Question 3: What are their barriers to using technology for health information?
- Question 4: Would technology be uniquely suited to address any specific information needs of older adults, and if so, what would those needs be?
- Question 5: What are the major medication information needs for older adults?
- Question 6: What kind of questions would an older adult ask to meet this information need?
- Question 7: What would be the overall pros and cons, hopes and concerns for this kind of project?

### Data Analysis

The transcribed interviews were analyzed using predetermined codes (Textbox 2). When important information was mentioned in an interview that did not correspond to an existing code, an ad hoc code was created to be reconciled later (eg, the code Need: Reminders was added by using this ad hoc process, representing the need for the system to give users medication reminders). For each interview, two of the four investigators (KR, ML, PR, and METR) coded according to the semiopen set of themes (Textbox 2). Of note, the coding scheme includes both a *benefit* and *nonbenefit*, as well as a *barrier* and *nonbarrier*. The negated codes were added because the interview participants frequently asserted that a particular benefit/barrier did not exist (eg, technology familiarity was not seen as a major hurdle for adults aged 55 to 75 years, so this was coded as Nonbarrier: Technology Familiarity/Assistance).

**Codes representing a set of themes.**

**Benefits/nonbenefits**

- Usability
- Support overall health
- Increased understanding
- Increased adherence
- Reduced adverse events

**Barriers/nonbarriers**

- Usability
- Technology familiarity/assistance
- Cost/affordability
- Trust in technology
- Difficulty hearing
- Cognitive ability/mental status
- Privacy and security

**Needs**

- Usability
- Reminders
- Indication
- Contraindication
- Instruction/dosage
- Adverse reaction
- Drug interaction
- Information

The codes were reconciled with the help of a third investigator (MG or SM). During this step, the ad hoc codes were considered, reconciled, and either included (if in use in at least 3 interviews) or dropped. Most of the ad hoc codes were used only once or twice, whereas other ad hoc codes were merged (eg, under the Usability need). This process was iterative and involved member checking with the interview participants. All interview participants were shown and agreed to the final interview descriptions.

The final codes for each interview were then counted for each category of benefit/nonbenefit, barrier/nonbarrier, and need. This count represents the number of times each interviewee’s response was directly mentioned or indirectly aligned with our themes. The initial subcategories did not include all the final lists, as shown in Textbox 2. The new codes include benefit: usability, support for overall health; barrier: usability, security; and need: usability, reminders, instruction, and information. The final counts for each interviewee in each category are presented in Table 1.

**Table 1:** Number of times each interviewee mentioned the preset themes in their responses.

Preset theme		Mention of preset theme, n							
		Expert 1	Expert 2	Expert 3	Expert 4	Expert 5	Expert 6	Expert 7	Expert 8
**Benefits**									
	Usability	1	2	0	0	1	4	2	4
	Support overall health	0	1	1	1	1	2	1	3
	Increased understanding	2	3	3	0	1	0	0	3
	Increased adherence	1	1	1	2	0	2	0	3
	Reduced adverse events	0	1	0	1	1	0	0	1
	Other benefit	0	0	0	4	2	0	0	1
**Barriers**									
	Usability	5	2	3	0	0	0	0	0
	Technology familiarity/assistance	2	2	1	5	5	2	1	4
	Cost/affordability	1	1	3	4	2	2	1	3
	Trust in technology	1	0	0	0	1	0	1	0
	Difficulty hearing	1	0	1	0	0	0	0	1
	Cognitive ability/mental status	0	0	3	0	1	0	0	0
	Privacy and security	2	5	1	0	0	0	0	3
	Other barrier	0	0	3	1	2	0	3	0
**Needs**									
	Usability	10	2	5	3	12	2	7	1
	Reminders	2	4	1	12	2	5	4	1
	Indication	2	2	2	1	2	2	2	0
	Contraindication	0	0	0	0	0	0	2	0
	Instruction/dosage	3	2	2	2	2	4	0	1
	Adverse reaction	2	1	3	4	3	1	1	1
	Drug interaction	1	0	1	3	2	0	1	0
	Information	3	1	2	4	0	0	0	2
	Other need	0	2	2	3	1	0	5	0
**Nonbenefits**									
	Overreliance on technology	0	0	0	0	1	0	0	0
	Other	0	0	0	0	0	1	0	0
**Nonbarriers**									
	Technology familiarity/assistance	3	3	2	3	4	1	2	2
	Technology familiarity/use	1	0	0	0	0	0	0	0
	Cost/affordability	2	0	0	0	0	0	0	0
	Trust in technology	1	0	1	0	0	0	0	0
	Cognitive ability	1	0	0	0	0	0	0	0
	Other	0	0	1	0	1	0	0	0

## Results

We have only focused on the aggregate results of the qualitative analysis in this section for brevity. We have provided a separate supplement that has detailed summaries of each of the 8 interviews and key quotes that illustrate each of the experts’ unique perspectives as well as their individual qualifications.

### Overall Common Themes

After aggregating the feedback from all interviews (Table 1), we have described the top 3 subcategories under each category, which were deemed important for designing or implementing the use of the medication information voice chatbot.

### Benefits

With regard to benefits, the most significant benefit would be related to usability. Being voice-based and having met most of the needs, a chatbot would be deemed very useful. Its ease of use; access to information; not having to type or see small print; and being connected to the pharmacy, health care providers, and their caregivers are some of the benefits categorized under usability. The next benefit would be that older adults would have increased knowledge and understanding of their medications by using a chatbot. Other equally beneficial aspects include increased adherence to medications and support for overall health.

### Barriers

Some of the most important barriers were related to technology familiarity and assistance. Overall, older adults from lower socioeconomic backgrounds and those who are very old (≥75 years) might have difficulty with technology. Next, the cost and affordability of such technology could be problematic, which would be mitigated if it were covered by insurance (eg, Medicare). The final concern was regarding privacy and security. Many older adults were not comfortable with devices listening to their conversations and may have been confused as to where their information could be sent or used.

### Needs

Among all the needs, having a voice-based chatbot that is usable (easy to use and useful) was deemed the most important (Textbox 3). Having the chatbot remind patients regarding medications, appointments, or refills was the next important need. Finally, information about adverse reactions and instructions to take medications were noted as equally important by our interviewees.

**Usability: components for each category.**

**Benefits**

- Voice adaptation
- Easy to use than other apps which require typing or seeing
- Easy access to information

**Barriers**

- Difficult to use
- Complex language
- Learning curve with different format of apps
- Failure to troubleshoot errors
- Voice recognition accuracy
- Usable only for a spectrum of population
- Inaccurate interoperability of chart among providers

**Needs**

- Ease of use
- Simple language and native language support
- Audible
- Technical support and troubleshooting errors
- Connect to personalized information
- Integration with existing devices
- Connect with pharmacy, physician and caregiver
- Disease-specific medication information, pronounce medication, track list of medications

### Nonbenefits

Only 2 interviewees mentioned the nonbenefits of the chatbot (ie, specific assertions that a potential benefit would not be realized). One expert was concerned about overreliance on technology, whereas another suggested that many older adults would not use it after the setup was completed by family.

### Nonbarriers

In contrast to what many may think that older adults are not familiar with technology, the experts largely agreed that for the age range we are focused on, this is actually not a problem. Technology familiarity and assistance emerged as one of the most important nonbarriers among older adults when using voice-based technology. This is especially noted in higher socioeconomic groups with access to and experience with using technology. They were also more likely to be in their 60s and have well-connected families and younger generations who helped them catch up on technologies.

## Discussion

### Principal Findings and Comparison With Prior Work

Our study explored the benefits, barriers, and needs of using a voice-based chatbot to address the medication information requirements of older adults. To gain insights, we conducted semistructured interviews with experts in geriatrics. Our experts’ feedback regarding chatbots and older adults aligns with previous study results: they could be useful overall, help older adults take care of themselves, and should be flexible to meet all older adults’ technical skills [9,11]. Our analysis of their feedback identifies many pointers that clear certain misconceptions regarding technology use in older adults and provides insight into the prominent aspects of implementing medication information chatbots. The most important aspect was that chatbots could be used by many older adults, and that technology familiarity is not a barrier that would have been expected.

The use of a medication information chatbot would benefit many older adults. The first and foremost benefit would be that voice-based chatbots help overcome many aging issues, such as diminished vision, tactile and dexterity issues, and patients with arthritis who cannot type. This in itself will give chatbots a benefit over a non–voice-based smartphone app that requires typing or looking up for information. In fact, age-related changes, such as fine motor skills, vision, hearing loss [22], and osteoarthritis [23], were found to be barriers to technology use in older adults. By being voice-based, these barriers can be addressed by making it easier to access medication information. However, the chatbot would have to be audible and come with a range of volume controls for older adults with hearing loss issues.

Next, if the chatbot could be connected with the pharmacy, providers, and caregivers, it would be very beneficial for older adults, as it would lower the burden of independently keeping track of medication lists. With the help of frequent reminders, older adults can have better medication adherence. Having access to knowledge would in turn lead to increased patient knowledge regarding health in general. It would also make them more independent in taking care of themselves, requiring fewer nursing homes or assisted living arrangements.

Having an interactive voice-based technology would mean more socializing for older adults who live alone, resulting in more use in some cases. Older adults use technology to socialize in terms of using it for calling or emailing [22]. In particular, with voice-based interactive technology, older adults reported feeling that they had a connection with it or felt less lonely, and some even quoted as it had become a friend [24]. Other benefits include increased peace of mind for caregivers, reduced chances of errors and a safety net, and sometimes helping with reminders for activities of daily living and prescription refills. The use of conversational agents in older adults after hospital discharge has been previously studied [25]. Their findings align with our experts’ feedback and suggest that having a bot integrated with telemedicine in such a patient population would benefit in supporting their health, as they could help them understand medical information and read out discharge instructions. Overall, older adults would find them easier to use compared with other mobile health apps, as they are age-friendly.

Technology familiarity may not be a significant barrier, according to our experts. In fact, many older adults aged ≤75 years were found to use some sort of technology for daily living, such as smartphones or computers. Similar findings were reported in a study performed in older adults. They participated in a focus group and voiced more positive attitudes toward technology than negative attitudes [26]. Another study mentioned that more than 50% of their older adult respondents reported using technologies such as smartphones and computers, whereas a lesser percentage used tablets [22]. These results contradict the stereotype that older adults might not favor the use of technology. Our study builds on this prior work by focusing on a high-value AI—a voice-based chatbot for medication information.

Our interviewees highlighted that older adults who are relatively younger (in their 60s) and have relatively high socioeconomic status have had experience with technology. Those who used or were exposed to technology while they worked and who were close to family members (especially younger generations) were likely to be comfortable using technology in general, or voice-based technology in particular. Many adults already use apps such as Alexa and Siri in their day-to-day activities. For such older adults, technology familiarity/assistance/use would not be a major barrier. However, for adults aged ≥70 years, those from lower socioeconomic status, and those who live alone or have weak family connections, technology familiarity could act as a barrier. Some of these factors can be overcome by making the chatbot affordable, making it easy to use, and helping with the setup process. Similarly, results from a more general study of older adults and barriers to technology use for daily living activities showed similar themes for barriers, such as educational limitations and limited access to technology [23].

Some of the other barriers put forth by the experts include concerns regarding cost and affordability. It might help integrate the chatbot into an app or already existing device to make it more affordable, as well as the fact that a stand-alone app may not be as highly used. Another option was to cover the cost of the chatbots through health insurance. The cost barrier for purchasing technology and device maintenance has been highlighted previously [23,27]. Security and privacy concerns were the next set of barriers. Many interviewees stated that older adults were skeptical about technologies that overheard their conversations and used that information to reach out for advertising. They were also confused about how and where their information would be shared. These concerns were more prominent among adults with paranoia, dementia, and mild cognitive impairment. Security and privacy concerns, along with data management confusions, were identified as key barriers in other studies as well [28,29]. Concerns related to how easy the chatbot would be for older adults to set up and use by themselves were also raised. For the chatbot to reach a wide variety of adults, experts believed that the technology must be extremely user-friendly and easy to use. This aligns with the results from a focus group conducted on older adults who were asked to provide feedback after using a tablet. They mentioned some of the barriers that were directly or indirectly related to the usability of such technologies, such as lack of instruction and guidance to use, lack of knowledge, and too much or too complicated technology [27]. Overall, even though certain barriers exist for the use of technology, our experts believe that a voice-based chatbot could be considered by many older adults, which aligns with the generally positive outlook noted in other studies [27,28].

To design and create a medication information chatbot, our experts suggested many pointers that could be essential needs expected of a chatbot. The most important being a usable chatbot. Many features were combined under usability (Textbox 3), such as the following specific suggestions: *ease of use of technology, easy setup, technical support and troubleshooting, simple language, native language support, has to be audible, personalized, useful for caregivers, can repeat back question, multiplatform, connected to personalized information, integrated with other existing devices, disease-specific medication information, integration with pharmacy, collaborate with provider, pronounce medication, ability to intake and store patient’s information, adverse reaction information, adverse reactions only mentioned when asked, track list of medications, and prompts family about refills due.*

The importance of having a usable technology to ease adaptability was reported earlier [28] in a focus group conducted on older adults. They mentioned that they would be frustrated with navigating through the technology or setting it up and felt that sometimes technologies made their life more difficult if they were not made simple to use. Some of their suggestions included simple instructions along with fewer buttons. Interestingly, they felt that speech-activated tools would be simpler for their age group. Training older adults was considered an option to overcome the usability barrier. Some of the older adults who were trained to use Alexa [24] reported that the training process made their adaptation to technology easier. Some of the components discussed under usability (Textbox 3), such as integration with pharmacies and collaboration with providers, were also considered important by older adults [13]. Moreover, the idea that the chatbot should mention adverse reactions only when asked to mimic the concerns expressed by some older adults who did not want to fully understand the side effects for the fear of change in their attitude toward taking the medications [14]. As caregivers were found to be very crucial in managing older adults’ overall health after discharge from the hospital [15], having a chatbot with the ability to connect to caregivers and prompt them regarding older adults’ medication refill needs would be useful.

Apart from a usable chatbot, it would require assisting adults with reminders, such as medication refill reminders, clinician appointments, and reminders about general health, such as checking blood pressure or blood sugar levels. Information about adverse reactions and instructions, as well as the dosage and timing of medication administration, were also important requirements for a chatbot functionality. This aligns with older adults’ expectations of a personal health app [13] and their medication information needs upon discharge from the hospital [14]. Similar needs were expressed by adults in a study on the use of chatbots for hypertension medication management [30]. This study included 33% of the adults aged >65 years. Their needs included having the ability to have medication lists, ability to set reminders, medication information and side effects, refill reminders, and integration with pharmacy and autorefill capacity. They also believed that having the chatbot integrated with a patient portal and being able to connect with the care team via a chatbot could help them update health data, such as blood pressure and weight. Most of them also wanted their chatbot to be personalized and being available on their phones.

To help with reminders, a chatbot would need patient-specific information that could be entered by the patient or caregiver, or received electronically. The latter would be favored because, from our analysis, having an easy-to-use chatbot would also reduce the manual tasks of entering information. For medication-specific information, it would require accessing data such as side effect resources to answer questions about adverse reactions [31] or extracting information from the Food and Drug Administration–mandated drug labels using natural language processing [32,33]. Such functionality can be used to answer questions about adverse reactions, drug interactions, and general information about the drug.

Many studies assert that the chatbot would be broadly accepted if it integrated with already existing technology and had multiple functionalities other than helping with medication administration or providing medication information. Some of them also suggested piloting this voice-based technology as an app on a smartphone. These features were viewed as highly important by older adults as well [30]. Our experts also suggested that they could be integrated into either home smart speakers (easy for older adults already using Alexa, Google Home, or Siri) or smart pill dispensers. Older adults felt that home smart speakers [34] were much simpler to use and were very impressed with its range of functionalities. Integrating medication information voice assistants into such systems might increase its adoption rates. According to an infographics report by eMarketer [35] in 2018, 7.3% of the population aged ≥65 years would have used a smart speaker device and its use would see a huge increment from use in 2017 (36.3%). This suggests that integration with smart speakers would benefit a large patient population.

Apart from discussing the benefits and barriers, our experts also emphasized some nonbenefits (potential benefits unlikely to be realized) and nonbarriers (ie, potential barriers they did not think would be problematic). Nonbenefits were few but included overdependence on technology and ignoring the chatbot. Older adults expressed fear of excessive reliance on technology in a pilot study [34] on their interaction with Google Home (voice assistant). Their specific concerns were the possible loss of creativity and less physical and mental exercise with the use of such agents. The other nonbenefit was that some older adults ignored the chatbot. This was expected to occur if the chatbot was not set up out of their own interest (eg, their family had set it up for them without consultation). Some older adults mentioned similar attitudes [28] wherein they were given smartphones by their family members (thinking older adults would find it a useful tool), but they never knew how to use it or what to do with it, and therefore never used it.

The significant nonbarrier derived from our analysis was technology familiarity and use, as discussed above. Other nonbarriers include trust in technology and age. Our experts mentioned that many older adults already use technologies, such as smartphones or computers, and some even use voice-based technologies such as Alexa or Siri in their daily lives. This translates to the idea that many older adults trust technology and have no inhibitions to share their information while using it. Another nonbenefit is age, as one of our experts mentioned that some of the older adults are more tech-savvy regardless of their age. However, as per the Pew Research Center [36], even though technology use has been on the rise among older adults in general, adoption and use declines above the age of 70 years when compared with ages 65 to 69 years. The adoption and use of certain technologies, such as smartphones, was seen to be higher among affluent, well-educated, and younger populations. Perhaps when other factors are considered along with age, someone who is affluent and older (above 70 years) might use technology more than someone who does not match the affluence scale but is younger. Another survey [37] was that apart from overall lesser adoption among older adults aged >75 compared with younger older adults, there was also a difference in the type of technology that older adults aged >75 used more than their younger counterparts, such as desktops and e-readers. These findings suggest that there might be differences in the adoption rates of voice-based medication information chatbots based on the older adults’ age and other factors, such as education and income levels.

### Limitations

Our needs analysis had several limitations. First, as our interviewee group was a small (8 experts) convenience sample, this may have led to a chance of bias. For instance, each of the interviewees was based in urban areas and a large part of a university-based hospital system. We originally planned to combine these interviews with a simulation study with older adults, but the COVID-19 pandemic prevented any use of a realistic simulation environment. Therefore, the identification of important needs from the adults’ perspective was missed, limiting our analysis to only geriatrics experts. We plan to conduct such a simulation-based study to supplement the findings of this study once the pandemic allows such a study to be conducted safely, specifically incorporating a diversity of patients across racial, ethnic, and socioeconomic groups. Second, as described above on the issue of usability, it is highly possible for 2 systems with highly similar sets of features to diverge greatly in their usability based on a small number of traits, which means that the overall perception of a system (as with any interactive system) is highly system-specific. Thus, it would be appropriate to repeat this study with a more specific focus on a specific medication information chatbot. Third, our interview participants were asked to focus on medication information needs for relatively recently prescribed medications (within 6 months). However, the medications that the patient has been taking for a long time are still associated with information needs, and these likely diverge from those of recently prescribed medications.

### Conclusions

A medication information chatbot would have an advantage in helping older adults with their medications, especially with reminders, instructions, increasing knowledge, and medication adherence. Even though technological capabilities would seem to be a barrier, most older adults are sufficiently familiar with technology, especially those from higher socioeconomic populations and adults who are close to younger generations. For the chatbot to be useful across a broad spectrum of older adults, designing an affordable chatbot that is easy and usable with troubleshooting capabilities, as well as connected with providers and pharmacies, would be of high priority. Usability has emerged as a significant factor, both under the need to construct a chatbot and the benefits of a chatbot. These findings suggest a framework for a voice-based, AI-powered medication information chatbot, although many of the findings require further investigation. Future work should dive deeper into identifying technological solutions to the particular needs and barriers that older adults face regarding medication information.

## Abbreviations

AI: artificial intelligence
IA: intelligent assistant
